# Combining bioinformatics and machine learning algorithms to identify and analyze shared biomarkers and pathways in COVID-19 convalescence and diabetes mellitus

**DOI:** 10.3389/fendo.2023.1306325

**Published:** 2023-12-19

**Authors:** Jinru Shen, Yaolou Wang, Xijin Deng, Si Ri Gu Leng Sana

**Affiliations:** ^1^ The First Clinical Medical School, Harbin Medical University, Harbin, China; ^2^ Department of Anaesthesiology, The Second Affiliated Hospital of Harbin Medical University, Harbin, China; ^3^ Department of Anaesthesiology, The First Affiliated Hospital of Harbin Medical University, Harbin, China

**Keywords:** COVID-19 convalescence, diabetes mellitus (DM), differentially expressed genes (DEGs), gene ontology (GO), protein-protein interaction (PPI), hub gene, machine learning

## Abstract

**Background:**

Most patients who had coronavirus disease 2019 (COVID-19) fully recovered, but many others experienced acute sequelae or persistent symptoms. It is possible that acute COVID-19 recovery is just the beginning of a chronic condition. Even after COVID-19 recovery, it may lead to the exacerbation of hyperglycemia process or a new onset of diabetes mellitus (DM). In this study, we used a combination of bioinformatics and machine learning algorithms to investigate shared pathways and biomarkers in DM and COVID-19 convalescence.

**Methods:**

Gene transcriptome datasets of COVID-19 convalescence and diabetes mellitus from Gene Expression Omnibus (GEO) were integrated using bioinformatics methods and differentially expressed genes (DEGs) were found using the R programme. These genes were also subjected to Gene Ontology (GO) functional enrichment analysis and Kyoto Encyclopedia of Genes and Genomes (KEGG) pathway analysis to find potential pathways. The hub DEGs genes were then identified by combining protein-protein interaction (PPI) networks and machine learning algorithms. And transcription factors (TFs) and miRNAs were predicted for DM after COVID-19 convalescence. In addition, the inflammatory and immune status of diabetes after COVID-19 convalescence was assessed by single-sample gene set enrichment analysis (ssGSEA).

**Results:**

In this study, we developed genetic diagnostic models for 6 core DEGs beteen type 1 DM (T1DM) and COVID-19 convalescence and 2 core DEGs between type 2 DM (T2DM) and COVID-19 convalescence and demonstrated statistically significant differences (*p*<0.05) and diagnostic validity in the validation set. Analysis of immune cell infiltration suggests that a variety of immune cells may be involved in the development of DM after COVID-19 convalescence.

**Conclusion:**

We identified a genetic diagnostic model for COVID-19 convalescence and DM containing 8 core DEGs and constructed a nomogram for the diagnosis of COVID-19 convalescence DM.

## Introduction

Severe acute respiratory syndrome coronavirus 2 (SARS-CoV-2), the virus responsible for coronavirus disease 2019 (COVID-19) worldwide pandemic, can cause mild to severe respiratory disease and non-respiratory symptoms (1). This virus can be rapidly transmitted, infection is associated with a relatively high mortality rate, and the virus can often evade the host’s immune response (2–4). Patient prognosis and long-term complications have become an increasingly important issue. In particular, an estimated 87.4% of patients who recovered from COVID-19 had at least 1 persistent symptom, especially in the neurological and respiratory systems (5).

Diabetes mellitus (DM) is a group of metabolic disorders characterized by chronic hyperglycemia that have multiple etiologies, all of which manifest as defects in insulin secretion and/or utilization. As of 2010, the global prevalence of DM in adults (20 to 79-years-old) was 6.4%, corresponding to 285 million cases. This prevalence was predicted to increase to 7.7% by 2030 (corresponding to 439 million adults), with the number of affected adults increasing by 69% in developing countries and by 20% in developed countries (6). DM is often divided into four categories according to its cause: Type 1 DM (T1DM), Type 2 DM (T2DM), gestational DM (GDM), and other types of DM (7). The destruction of pancreatic beta cells leads to the development of T1DM, and this type of diabetes often leads to an absolute deficiency of insulin. T2DM is characterized by insulin resistance, and decreased insulin secretion and decreased function of pancreatic beta cells may be the initiating factor in most cases. Although short-term hyperglycemia has no serious effects on the body, long-term hyperglycemia can lead to chronic changes, such as microvascular complications (e.g., diabetic nephropathy, diabetic retinopathy, and neuropathy) and devastating macrovascular complications, such as cardiovascular diseases, that can have irreversible and even fatal effects (8).

Many previous studies showed significant increases in the prevalence, severity, and mortality of COVID-19 in patients with DM compared to non-diabetic patients, suggesting an association of COVID-19 severity with poor glycemic control (9, 10). Other studies suggested that COVID-19 may predispose infected individuals to hyperglycemia and promote the development of DM (11, 12).

SARS-CoV-2 binds to angiotensin-converting enzyme 2 (ACE2), and this protein is expressed in the lungs and many other organs, including the pancreas (13). This suggests that new-onset hyperglycemia and DM in patients with COVID-19 may be due to a direct attack of SARS-CoV-2 on islet β-cells in the pancreas. Therefore, it is crucial to identify the common biomolecules and pathways that are altered in patients with DM and patients undergoing convalescence following COVID-19. These shared biomolecules may have potential as biomarkers or therapeutic targets.

In this study, we used bioinformatics and machine learning algorithms to identify differentially expressed genes (DEGs) and predict altered molecular regulatory networks in patients undergoing convalescence from COVID-19, patients with T1DM, and patients with T2DM. Our findings may provide a basis for development of new measures that could be used for disease prevention and treatment in these patients.

## Materials and methods

### Acquisition of chip data

Data from the National Center for Biotechnology Information (NCBI) Gene Expression Omnibus (GEO) (https://www.ncbi.nlm.nih.gov/geo) were used to determine similarities of gene expression in patients undergoing COVID-19 convalescence (1, 3, and 6 months after hospital discharge), patients with T1DM, and patients with T2DM, with the search restricted to humans (**Table 1**). The GSE227116 dataset was generated by RNA sequencing (RNA-seq) of whole blood, and contains data from 75 samples: 65 patients after COVID-19 convalescence and 10 healthy donors. In particular, the study group was the population who had already recovered from COVID-19 infection. This sample was selected for follow-up analysis to investigate the long-term alterations after COVID-19 convalescence. For analysis of T1DM, three microarray datasets of peripheral blood mononuclear cells (PMBCs; GSE193273, GSE29142, and GSE55098) were selected (**Table 1**). GSE193273 contains data from 20 T1DM patients and 20 healthy controls; GSE29142 contains data from 9 T1DM patients and 10 healthy controls; and GSE55098 contains data from 12 T1DM patients and 10 healthy controls (**Table 1**). Similarly, for analysis of T2DM, three microarray datasets of PMBCs (GSE163980, GSE156993, and GSE9006) were selected; these data consist of gene expression data from 29 T2DM patients and 35 healthy controls.

**Table 1:** Training and validation datasets used for analysis.

Clinical status	Accession No.	Source	Platform	Cases : Controls
COVID-19 convalescence	GSE227116	Whole blood	GPL16791	65:10
Training				
T1DM	GSE193273	PBMCs	GPL20844	20:20
T1DM	GSE29142	PBMCs	GPL13507	9:10
T1DM	GSE55098	PBMCs	GPL570	12:10
T2DM	GSE156993	PBMCs	GPL570	12:6
T2DM	GSE163980	PBMCs	GPL20115	5:5
T2DM	GSE9006	PBMCs	GPL96	24:12
Validation				
T1DM	GSE33440	PBMCs	GPL6947	16:6
T2DM	GSE41762	pancreatic islets	GPL6244	20:57
COVID-19	GSE166253	PBMC	GPL20795	6:10

PMBCs, peripheral blood mononuclear cells; T1DM, type 1 diabetes mellitus; T2DM, type 2 diabetes mellitus.

### Data processing and differential expression analysis

For the dataset of COVID-19 convalescent patients (GSE227116), the limma package in R software(version 4.3.0) was used to identify changes in gene expression (fold change ≥ 1.2, |log_2_(FC)| ≥ 0.263) (14, 15). All *P-*values were adjusted using the Benjamini-Hochberg correction, and the false discovery rate (FDR) threshold for DEGs was 0.05. Due to factors such as theoretical approximations, methodological difficulties, limitations in the sensitivity and resolving power of experimental instruments, instability of the surrounding environment, limitations in the observer’s ability to discriminate between senses, and variability in technical proficiency, there will always be a deviation between the measurement results and the true value of the measurement. The problem of measurement error is equally present in this study. For analysis of the T1DM and T2DM datasets, the ComBat package was first used to process the gene expression data to eliminate batch effects (16). Then, the differentially expressed genes were obtained using the limma package in R software, as described above, and a heat map was generated (17).

### Gene ontology and pathway enrichment analysis

The Gene Ontology (GO) and Kyoto Encyclopedia of Genes and Genomes (KEGG) databases were used to analyze gene-related functions. The GO database classifies gene function as cellular component (CC), molecular function (MF), and biological process (BP) (18), and the KEGG database provides information about the related pathways. The Clusterprofiler package in R software was used for subsequent analysis and identification of information about gene function and potential pathways (19). The conditional filtering used a *P*-value cutoff of 0.05, the ggplot2 package in R software was used for visualizing BP in the GO enrichment analysis, and the graph package in R was used for visualizing the KEGG pathway.

### Protein-protein interaction network analysis

The filtered DEGs were uploaded to the Search Tool for the Retrieval of Interacting Genes/Proteins (STRING) database (https://string-db.org/) (20). The minimum required interaction score was set at 0.4 (medium confidence), and the disconnected nodes were hidden to construct a protein-protein interaction (PPI) network. Cytoscape software was used for network display, layout, and query, to integrate the biological networks and molecular information (such as gene expression and genotype) in a visual environment, and to link these networks with functional annotation databases (21). The resulting PPI networks were imported into Cytoscape software version 3.9.1, and the DEGs were ranked and filtered using the Matthews correlation coefficient (MCC) algorithm.

### Transcription factors and miRNAs

To explore the potential impact of core DEGs on subsequent molecular regulatory mechanisms, predicted transcription factors (TFs) and miRNAs from databases were obtained from the networkanalyst (https://www.networkanalyst.ca/). These data were from JASPAR, an open source database of TF binding sites in the form of position frequency matrices (PFMs) and TF flexible models (TFFMs) that record DNA binding preference information of TFs in six major species (22). This database identified topologically credible TFs. The TF-gene relationships were then imported into Cytoscape software version 3.9.1 to construct a visual regulatory network.

The miRTarBase database is a specialized collection of microRNA-mRNA targeting relationships (MTI, MicroRNA-Target Interactions), and all of its data were experimentally validated (23). This database was used to obtain DEG-associated miRNAs. miRNAs were retained if they interacted with more than two DEGs for construction of a visual miRNA-gene interaction network.

### Construction and validation of a diagnostic DEG signature

Two machine learning algorithms were used to screen for shared changes in biomarkers in the two pair-wise comparisons (COVID-19 convalescence + T2DM, COVID-19 + T1DM). Elastic Net Regressions is a regularization algorithm that combines the features of Lasso regression and ridge regression with the advantages of sparsity and variable selection. When multiple features are related, Lasso regression may only randomly choose one of them, ridge regression can choose all of the features. By combining these two regularization methods using elastic net regressions, we are able to bring together the strengths of both methods (24). The shrinkage regularization parameter λ, which controls the complexity of the model, was determined by 10-fold cross-validation of the partial likelihood deviance and the attendant ‘1 standard error rule’. The elastic net used 10-fold cross-validation and fitted a linear model using a penalty score (α = 0.9). The elastic net algorithm for variable reduction and selection utilized the glmnet package in R (25); the independent variables were the normalized expression matrix of DEGs, and the dependent variables were the presence or absence of disease in the sample using a 10-fold cross validation. Support vector machine-recursive feature elimination (SVM-RFE) was used to identify the optimal hyperplane that partitioned the training dataset and maximized the geometric interval (26). This calculation used a 5-fold cross validation within the e1071 package in R (27). The top-ranked DEGs were selected from PPI network identification and the intersection of the two machine learning algorithms. Then, column line plots and receiver operating characteristic (ROC) analysis with area under the curve (AUC) values were used to assess the model. The dataset was selected from an extensive search in the GEO database. The selection criteria for the DM validation set were as follows: (a) The datasets are all of the type of expression profiling by array. (b) Both contain patients with diagnosed diabetes and controls from healthy people. External validation datasets in GEO were used for model validation: GSE33440 (16 T1DM and 6 healthy individuals) and GSE41762 (20 T2DM and 57 healthy individuals). The COVID-19 convalescence validation dataset GSE166253 (6 convalescent patients, and 10 healthy controls) was obtained from an extensive search in GEO.

### Evaluation and correlation of immune cell infiltration

Single-sample gene set enrichment analysis (ssGSEA), a method commonly used for analysis of immune cell infiltration, was used to estimate the relative enrichment of a gene set in each sample by comparing the gene expression data of a sample with a specific immune cell gene set and to estimate the relative abundance of different immune cell types in each sample (28).The immune cell marker genes were from **Supplementary Table S1** in the study of Bindea et al., which provided information on 24 immune cells (29). The immune infiltration status of each sample was then obtained using ssGSEA for two hub DEGs (*CD3G* and *YES1*) in the T1DM and COVID-19 convalescence datasets, and two hub DEGs (*PTRF and EHD1*) in the T2DM and COVID-19 convalescence datasets.

## Results

### Removal of batch effects

**Figure 1:**
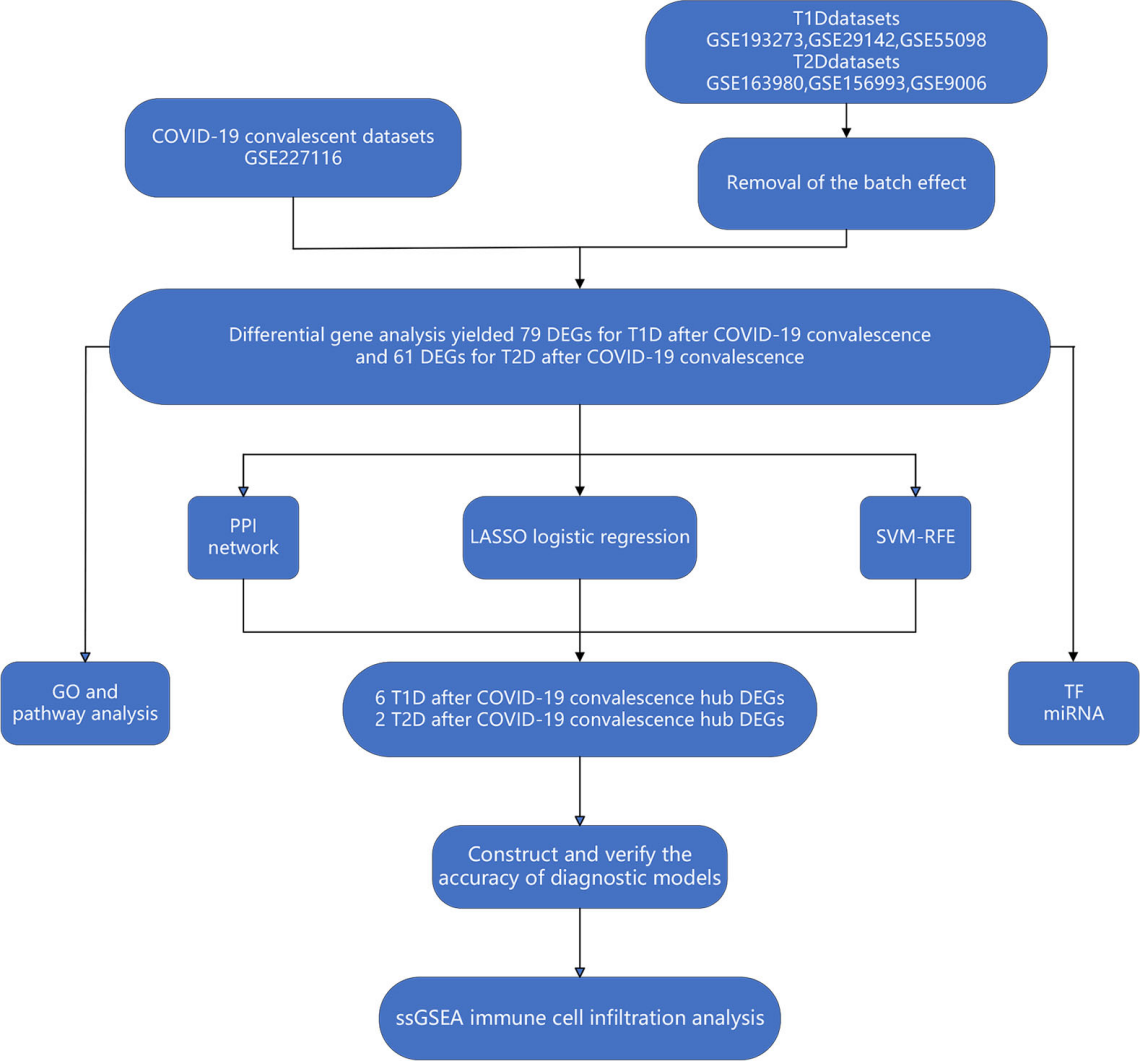
Workflow of bioinformatics and machine learning analyses used in the present study.

**Figure 2:**
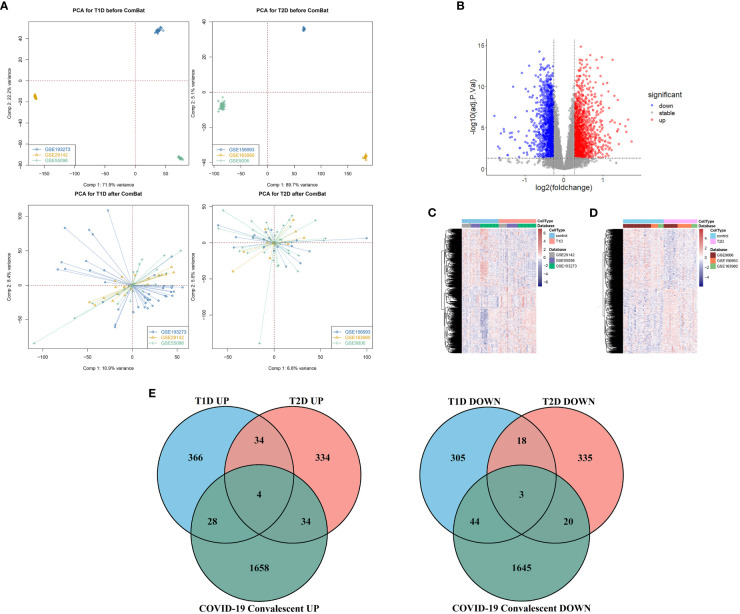
**(A)** Principal component analysis before removal of batch effects (top), and after removal of batch effects (bottom). **(B)** Volcano map of differentially expressed genes in the COVID-19 convalescence and the healthy population datasets. **(C)** Heat maps of differentially expressed genes in the T1DM and healthy population datasets. **(D)** Heat maps of differentially expressed genes in the T2DM and healthy population datasets. **(E)** Venn diagrams of shared differentially expressed genes from comparison of COVID-19 convalescence + T2DM, and of COVID-19 convalescence + T1DM.

Different datasets can have statistically significant differences in the expression of the same genes in patients with the same disease due to differences in experimental reagents, operators, processing time, laboratory equipment, and other factors (30). We therefore used the ComBat function in the sva package to remove these batch effects and achieve a convergence in the distribution of expression values for the different datasets (**Figure 1**). Principal component analysis (PCA) of the sample distributions in the three T1DM datasets and the three T2DM datasets before and after the elimination of the batch effect showed that this method was successful (**Figure 2A**).

### Similar DEGs in the different datasets

We analyzed 75 samples from the RNA-seq dataset (GSE227116) of controls and subjects with COVID-19 convalescence using the limma package in R software. There were 3436 DEGs, with 1724 up-regulated genes and 1712 down-regulated genes (**Figure 2B**). After removing batch effects, we used the same method for analysis of the three T1DM datasets and the three T2DM datasets. There were 81 samples in the T1DM datasets (41 cases and 40 controls), and the analysis identified 802 DEGs (**Supplementary Figure 1A**), with 432 up-regulated genes and 370 down-regulated genes (**Figure 2C**). There were 64 samples in the T2DM datasets (29 cases and 35 controls), and the analysis identified 782 DEGs (**Supplementary Figure 1B**), with 406 upregulated genes and 376 genes downregulated genes (**Figure 2D**).

We used Venn diagrams to compare the DEGs in the COVID-19 convalescence, T1DM, and T2DM datasets (**Figure 2E**). T1DM and T2DM contained 38 co-up-regulated DEGs and 21 co-down-regulated DEGs (**Supplementary Table 1**). The COVID-19 convalescence and T1DM data had 79 of the same DEGs (32 up-regulated genes and 47 down-regulated genes). The COVID-19 convalescence and T2DM data had 61 of the same DEGs (38 up-regulated genes and 23 down-regulated genes).

### KEEG enrichment analysis

**Figure 3:**
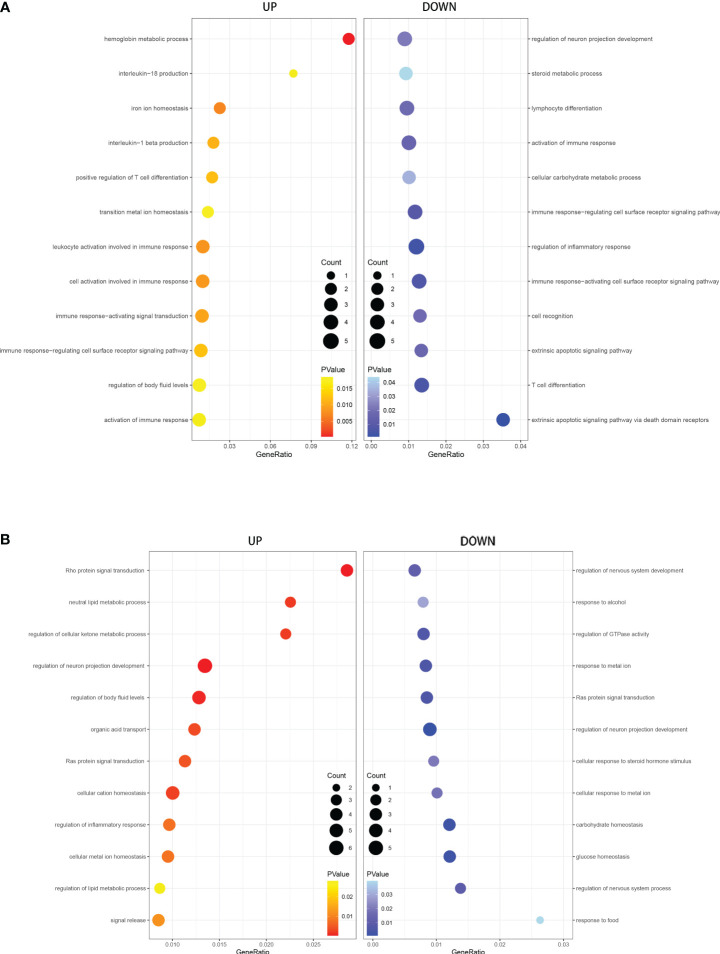
GO analysis of DEGs in T1DM and COVID-19 convalescence **(A)** and in T2DM and COVID-19 convalescence **(B)**, showing genes enriched in biological processes that were upregulated (left, red) and down-regulated (right, blue).

**Figure 4:**
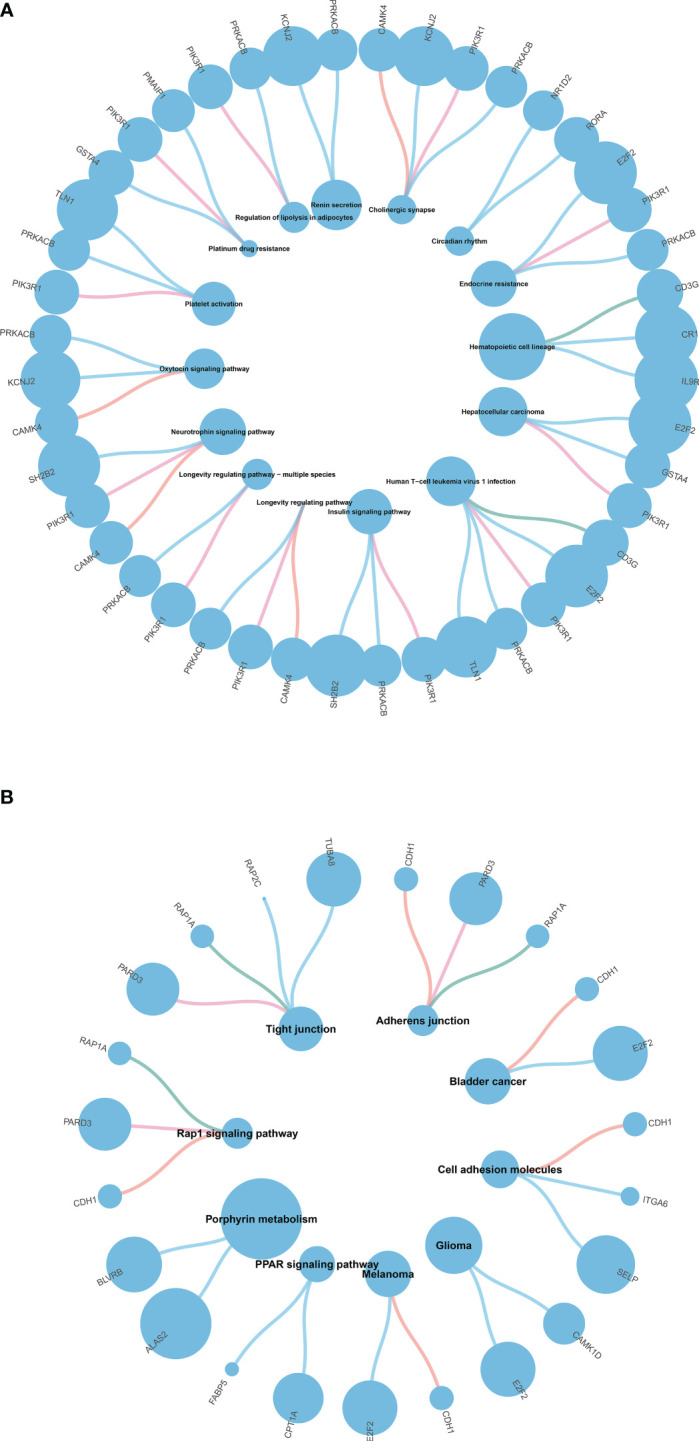
**(A)** KEGG pathway enrichment analysis of T1DM and COVID-19 convalescence **(A)** and T2DM and COVID-19 convalescence **(B)**.

We performed GO and KEGG enrichment analysis of the DEGs to identify the main biological processes and pathways in T2DM, T1DM, and COVID-19 convalescence. The upregulated genes in T1DM and COVID-19 convalescence were mainly clustered in “hemoglobin metabolic process”, “interleukin-18 production”, “iron ion homeostasis”, and “positive regulation of T cell differentiation”; the downregulated genes were mainly involved in “regulation of neuron projection development”, “steroid metabolic process”, “activation of immune response”, and “other biological processes” (**Figure 3A**, **Supplementary Table 2**). KEGG enrichment analysis demonstrated enrichment in “human T-cell leukemia virus 1 infection (HTLV-1)” and “Cholinergic synapse” pathways (**Figure 4A**).

The upregulated genes in T2DM and COVID-19 convalescence included “Rho protein signal transduction”, “neutral lipid metabolic process”, and “regulation of cellular ketone metabolic process”; the downregulated genes included “regulation of nervous system development”, “response to alcohol”, and “regulation of GTPase activity” (**Figure 3B**, **Supplementary Table 2**). KEGG enrichment analysis demonstrated enrichment in “Tight junction”, “Adherens junction”, “Rap1 signaling pathway”, and “Cell adhesion molecules” (**Figure 4B**).

### Construction of the PPI network

We then separately entered the 79 DEGs from comparison of the T1DM and COVID-19 convalescence datasets and the 61 DEGs from comparison of the T2DM and COVID-19 convalescence datasets into the STRING database to determine their relationships. The average node degree is the average value of the interaction of proteins in the network. It is used to measure the strength of the interaction relationship between proteins. For the first comparison (T1DM + COVID-19 convalescence), the PPI network contained 77 points, 21 edges, and the average node degree was 0.545 (**Figure 4A**). For the second comparison (T2DM + COVID-19 convalescence), the PPI network contained 61 nodes, 18 edges, and the average node degree was 0.59 (**Figure 4B**).

**Figure 5:**
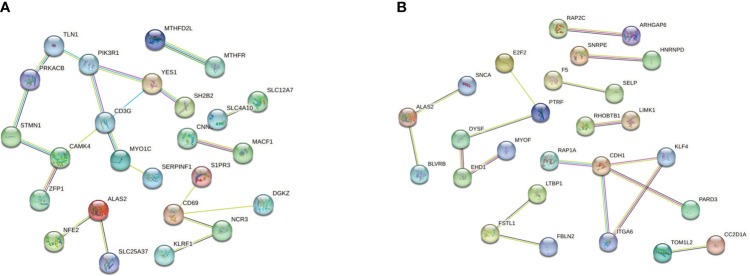
**(A)** PPI network analysis of T1DM and COVID-19 convalescence **(A)** and T2DM and COVID-19 convalescence **(B)**.

We then performed PPI network analysis using Cytoscape software with the cytoHubba plugin. Comparison of the T1DM and COVID-19 convalescence datasets indicated the 11 major DEGs were *CD3G*, *CAMK4*, *PIK3R1*, *YES1*, *CD69*, *ALAS2*, *STMN1*, *MYO1C*, *NCR3*, *TLN1*, *PRKACB* (**Figure 5A**) sorting by degree value. Comparison of the T2DM and COVID-19 convalescence datasets indicated the 8 major DEGs were *CDH1*, *ALAS2*, *KLF4*, *ITGA6*, *DYSF*, *PTRF*, *EHD1*, and *FSTL1* (**Figure 5B**) sorting by degree value. These are considered to be genes with a high degree of gene-gene interaction and they have a strong association with DEGs. These results suggest that future studies that focus on these DEGs may provide new therapeutic strategies for disease prevention or treatment in these patients.

### Determination of regulatory signatures

**Figure 6:**
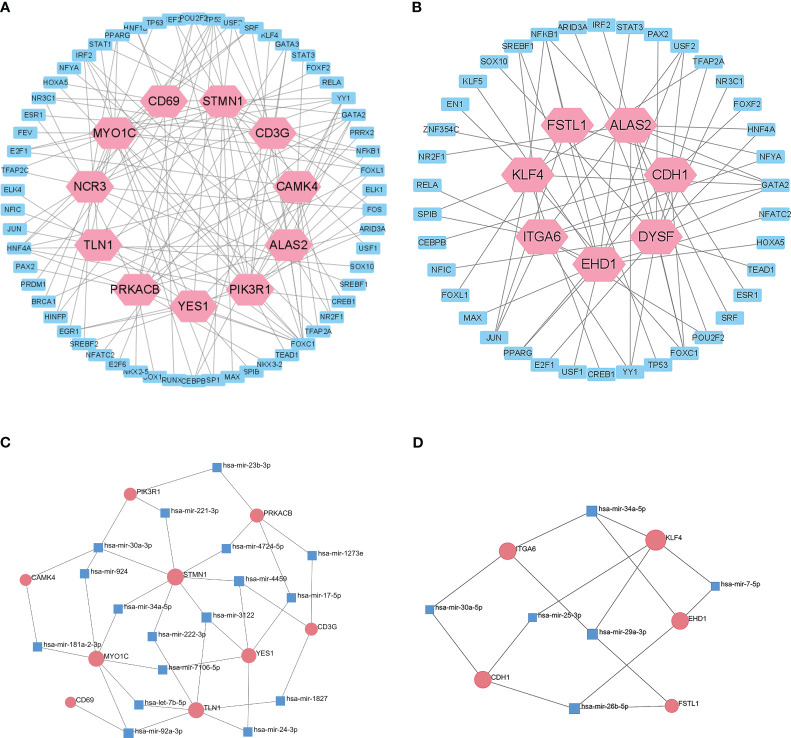
Interactions of DEGs with potential TFs of T1DM and COVID-19 convalescence **(A)** and T2DM and COVID-19 convalescence **(B)**, based on the JASPAR database. Interactions of DEGs with potential miRNAs of T1DM and COVID-19 convalescence **(C)** and T2DM and COVID-19 convalescence **(D)**, based on miRTarBase database.

We then used the JASPAR database to analysis the relationships of TFs in the different datasets (**Figures 6A, B**). Cytoscape identified 58 shared TFs in a comparison of the T1DM and COVID-19 convalescence datasets, and 38 shared TFs in a comparison of the T2DM and COVID-19 convalescence datasets. Analysis of gene-miRNA relationships using the miRTarBase database (**Figures 6C, D**) identified 17 shared miRNAs in a comparison of the T1DM and COVID-19 convalescence datasets, and 6 shared miRNAs in a comparison of the T2DM and COVID-19 convalescence datasets. Each of these miRNAs was associated with two or more DEGs.

### Construction of a prognostic model

**Figure 7:**
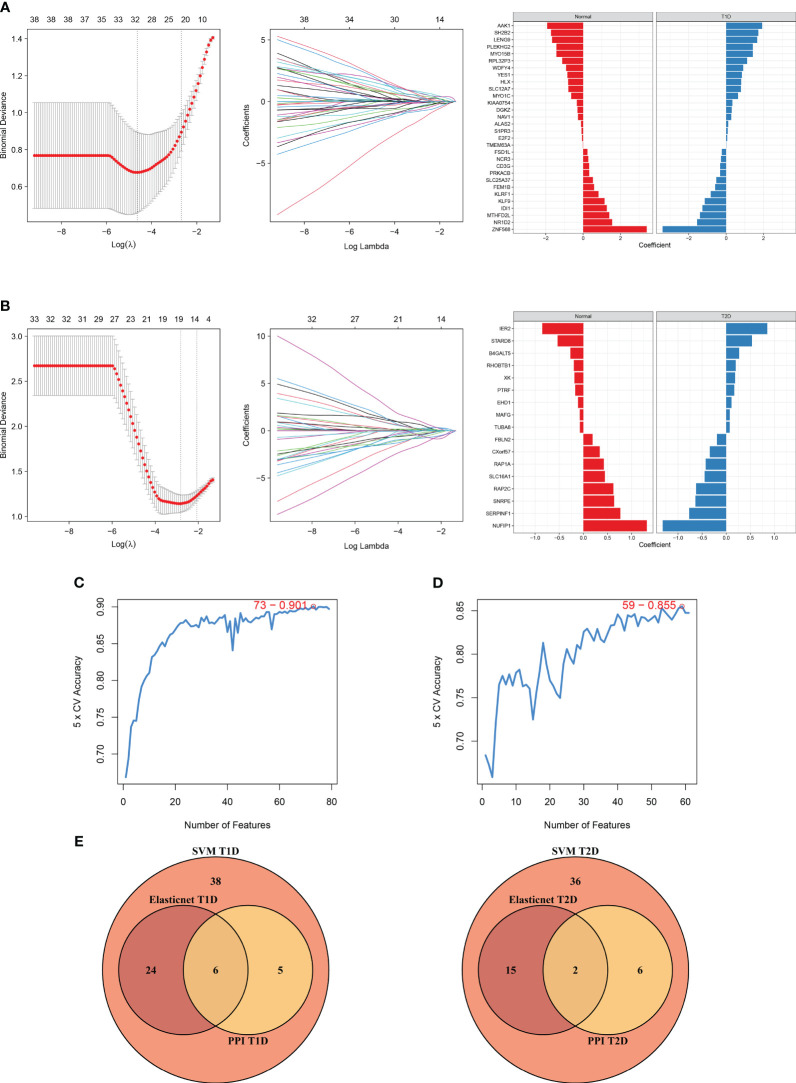
Diagram of the machine algorithm obtained by inputting overlapping DEGs. **(A)** Elastic Net Regression screening for shared diagnostic markers in T1DM and COVID-19 convalescence (left), identification of different genes by color (middle), and coefficient values of the resulting genes(right). **(B)** Elastic Net Regression screening for shared diagnostic markers in T2DM and COVID-19 convalescence (left), identification of different genes by color (right), and coefficient values of the resulting genes(right). **(C)** SVM screening of biomarkers with highest accuracy for T1DM and COVID-19 convalescence. **(D)** SVM screening of biomarkers with highest accuracy for T2DM and COVID-19 convalescence. **(E)** Venn diagrams hand over hub DEGs common to LASSO algorithm, SVM algorithm and PPI network MCC algorithm.

We also used elastic net regression to analyze the DEGs from the two comparisons. The penalty factor λ for the comparison of T1DM and COVID-19 convalescence was 0.01 (log(λ) = −4.54) and the regression identified 30 genes (**Figure 7A**). The penalty factor λ for the comparison of T2DM and COVID-19 convalescence was 0.06 (log(λ) = −2.81), and the regression identified 17 genes (**Figure 7B**). The input of SVM algorithm was 79 and 61 DEGs that were up-regulated or down-regulated at the same time in COVID-19 convalescence and T1/2DM, and other non-essential genes were not included. The results from the SVM algorithm (**Figure 7C**) showed that the highest accuracy (90.1%) was achieved at gene number 73 in the T1DM and COVID-19 convalescence datasets. The mean accuracy with standard deviation was 86.49 ± 4.72% (**Supplementary Table 3**, **Supplementary Figure 2**). This represents that after ranking the features and selecting the most important features for classification, when the number of genes is 73, its accuracy in high-dimensional space is the highest, which can effectively separate different categories in the datasets. The highest accuracy (85.5%) was at gene number 59 in the T2DM and COVID-19 convalescence datasets (**Figure 7D**). The mean accuracy with standard deviation was 80.21 ± 4.66% (**Supplementary Table 3**, **Supplementary Figure 3**). We then identified the intersection of genes screened by the two machine learning algorithms with the top-ranked genes of the MCC algorithm in the PPI network (**Figure 7E**). They are considered to be the most prominent hub DEGs affecting DM and COVID-19 convalescence. The results indicated six hub DEGs for comparison of T1DM and COVID-19 convalescence (*CD3G*, *YES1*, *ALAS2*, *MYO1C*, *NCR3*, *PRKACB*) and two hub DEGs for comparison of T2DM and COVID-19 convalescence (*PTRF, EHD1*).

**Figure 8:**
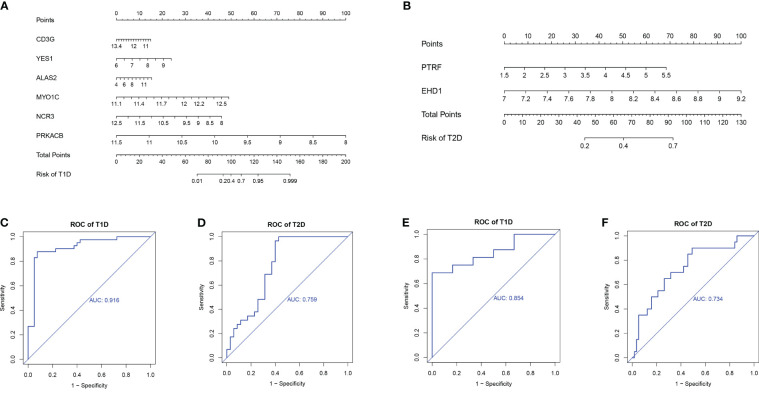
Nomograms for prediction of T1DM after COVID-19 convalescence **(A)** and T2DM after COVID-19 convalescence **(B)**. ROC curves for prediction of T1DM after COVID-19 convalescence **(C)** and T2DM after COVID-19 convalescence **(D)** using the training datasets. ROC curves for prediction of T1DM after COVID-19 convalescence **(E)** and T2DM after COVID-19 convalescence **(F)** using the validation datasets.

We used the rms package to construct column lineage maps of the signature genes from the two comparisons (**Figures 8A, B**), and used ROC curves to assess the performance of the prediction model. They are all based on combinations of training datasets. The AUC was 0.916 for prediction of T1DM based on COVID-19 convalescence data (**Figure 8C**), and the AUC was 0.759 for prediction of T2DM based on COVID-19 convalescence data (**Figure 8D**). We then used two other datasets (GSE33440 for T1DM and GSE41762 for T2DM) from the GEO database to construct ROC curves and validate the model. The AUC value in the validation analysis was 0.854 for prediction of T1DM data (**Figure 8E**) and 0.734 for prediction of T2DM data (**Figure 8F**). Similarly, we constructed predictive models with 6 hub DEGs for T1DM and 2 hub DEGs for T2DM in the COVID-19 convalescence validation set, both with an AUC value of 1.0 (**Supplementary Figure 4**).

### Correlations of hub DEGs and immune cell infiltration

**Figure 9:**
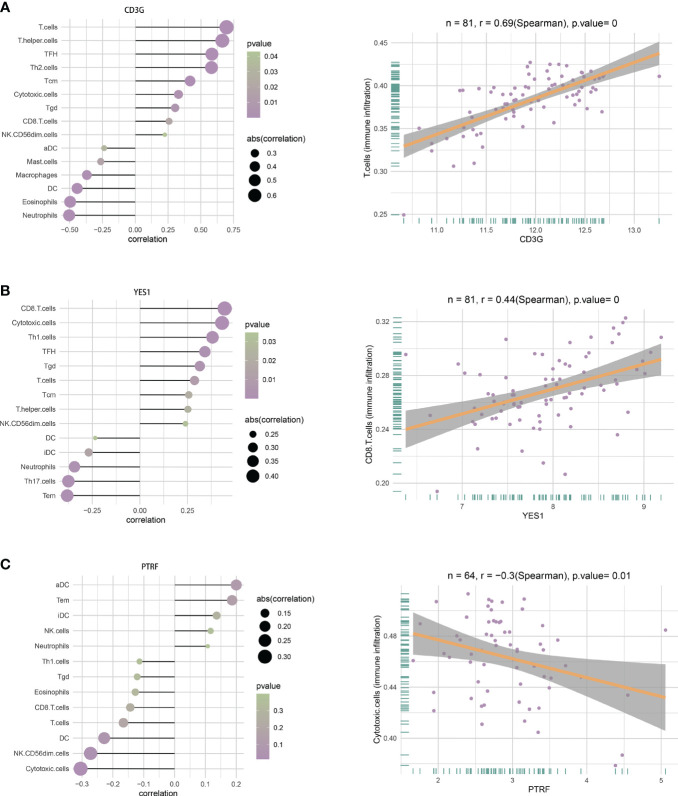
**(A)** Correlations between CD3G and infiltration of different immune cells (left), and between CD3G and T cells (right). **(B)** Correlation between YES1 and infiltration of different immune cells (left), and between YES1 and CD8 T cells (right). **(C)** Correlation between PTRF and the infiltration of different immune cells (left), and between PTRF and cytotoxic T cells (right).

We analyzed the relationship of immune cell infiltration with two hub DEGs (*CD3G* and *YES1*) in the T1DM and COVID-19 convalescence data, and with two other hub DEGs (*PTRF and EHD1*) in the T2DM and COVID-19 convalescence data. The results showed that the expression of CD3G had a positive correlation with the infiltration of T cells (r = 0.69, *p* < 0.01, **Figure 9A**) and the expression of YES1 had a positive correlation with the infiltration of CD8+ T cells (r = 0.44, *p* < 0.01, **Figure 9B**). PTRF expression had a negative correlation with cytotoxic cell infiltration (r = −0.3, *p* = 0.01, **Figure 9C**). EHD1 expression had a low correlation with immunocyte (r = 0.25, *p* = 0.05, **Supplementary Figure 5**).

## Discussion

COVID-19 continues to have a high worldwide prevalence, and a growing body of evidence indicates it can lead to pathophysiological changes in glucose metabolism. New-onset diabetes is the most common COVID-19 comorbidity, and these patients often experience a dramatic deterioration and poor prognosis (31). Therefore, identification of the genes and pathways that are altered after COVID-19 convalescence is essential for understanding the molecular basis of DM in these patients. In this study, we used a bioinformatics approach to identify potential biomarkers of new-onset DM in patients after COVID-19 convalescence.

We identified 79 of the same DEGs in whole blood samples of patients undergoing COVID-19 convalescence and in PMBC samples of T1DM patients, with 32 up-regulated genes and 47 down-regulated genes. We also performed KEGG enrichment analysis of these shared DEGs. GO analysis showed that the upregulated DEGs were mainly associated with hemoglobin metabolic processes, production of inflammatory substances, and homeostasis of metal ions, and the down-regulated DEGs were mainly associated with activation of immune responses and metabolism of *in vivo* substances. Previous research reported an increased responsiveness of lymphocytes in T1DM, and disruption of immune homeostasis is a major problem in diabetes, consistent with our findings (32). Our KEGG analysis showed that most of these genes were enriched in the HTLV-1 infection pathway and the cholinergic synaptic pathway. Although genes in the HTLV-1 infection pathway had the greatest enrichment, there is no experimental evidence that COVID-19 is significantly associated with HTLV-1. However, a clinical cross-sectional study reported that HTLV-1 patients with a high proviral load were more likely to develop DM and chronic kidney disease (33). Therefore, we speculate COVID-19 convalescence may activate the HTLV-1 infection pathway by altering T cells, thus promoting the development of DM. There is evidence that cholinergic synapses are altered in patients with different in neurological disorders, and that activation of the parasympathetic nervous system in the pancreas increases plasma insulin levels and improves glucose tolerance, but activation of the sympathetic nervous system in the pancreas has the opposite effect (34). In agreement, our results demonstrated that this pathway plays a crucial role in the pathogenesis of DM during COVID-19 convalescence.

These results led us to construct a PPI network and use two machine learning algorithms to identify diagnostic biomarkers that were present in T1DM and COVID-19 convalescence: *CD3G*, *YES1*, *ALAS2*, *MYO1C*, *NCR3*, and *PRKACB*. Among these genes, *CD3G* and *YES1* interacted with most of the other genes. More specifically, these two genes were both down-regulated in COVID-19 convalescence and DM, and we identified them as the most important hub DEGs. *CD3G* functions in T cell activation, signaling, and regulation of T cell receptor (TCR) expression, and defects in this gene result in a defective T cell response to mitogenic signals (35). Several recent bioinformatics studies found that *CD3G* is closely associated with tumors, such as cervical cancer and triple-negative breast cancer (36, 37), and with immune system alterations that occur during Sjögren’s disease (38). *CD3G* is an isoform of the T cell transmembrane protein CD3 antigen, and occurs as a CD3G/CD3E heterodimer, which forms a TCR-CD3 complex with the alpha and beta chains of the TCR. Specific MHC peptide complexes that are produced by antigen presenting cells (APC) can form complexes with TCR-CD3 and induce activation of T cells. Down-regulation of *CD3G* can lead to compromised immune function, and may induce a variety of autoimmune diseases (39).

*YES1* is a non-receptor tyrosine kinase that functions in GLUT4-mediated glucose transport and is in the SRC family of kinases (SFK). The SFK regulates a variety of cellular processes and has an important role in maintaining cellular homeostasis. The unique serine/threonine phosphorylation domain in *YES1* regulates cell cycle progression, and this gene has high expression in a variety of tumors, including non-small cell lung cancer (40), gastric cancer (41), ovarian cancer (42), and breast cancer (43), and is therefore considered a novel therapeutic target and biomarker for cancer (44). *YES1* is associated with several receptor tyrosine kinases (RTKs; EGFR, CSF1R, and SCFR), G protein-coupled receptors (P2RY2 and AT1R), and cytokine receptors (IL11, CD95, and GM-CSF). Previous research demonstrated that YES1 acted as a proximal glucose-specific activator of cell division cycle control protein 42 (Cdc42) in pancreatic islet cells, and therefore affects insulin secretion. Cdc42 is a small GTPase in the Rho family, and there is evidence that it is the proximal glucose-specific trigger of insulin secretion and that its activation of downstream signals ultimately leads to mobilization of insulin granules to the plasma membrane (45).

Forkhead-box C1 (*FOXC1*) is a TF that regulates the expression of *CD3G* and *YES1*, and may therefore affect the development of T1DM after COVID-19 convalescence. Previous studies reported that *FOXC1* increased glucose uptake and improved insulin sensitivity and had a role in the pathogenesis of GDM by attenuating the high-glucose (HG)-induced trophoblast damage by upregulating the FGF19-activated AMPK signaling pathway (46).Our search for miRNAs that bind to CD3G and *YES1* led to the identification of hsa-mir-4459. Previous studies suggested that this miRNA may function in the photodynamic therapy (PDT)-induced apoptosis of glioma cells. We suggest that *FOXC1* and hsa-mir-4459 have potential as key biomarkers or therapeutic targets for treatment of T1DM after COVID-19 convalescence.

We used the same approach to compare T2DM and COVID-19 convalescence. The enrichment analysis demonstrated 38 shared upregulated genes and 23 shared downregulated genes, and GO analysis showed that the upregulated genes were mainly associated with Rho protein signaling. Rho kinase (ROCK) is a serine/threonine protein kinase that is activated by binding to RhoA, and the RhoA/ROCK pathway regulates cell contraction, migration, adhesion, proliferation, and inflammatory responses (47). There is evidence that ROCK interacts with the insulin receptor substrate-1 (IRS-1) and impairs insulin signaling in skeletal muscle, and that the resulting increased insulin resistance leads to the development of T2DM (48, 49). ROCK inhibitors therefore have great potential for treatment of diabetes and its complications (50). Our findings are thus consistent with these previous results.

Down-regulated genes in T2DM and COVID-19 convalescence were enriched in the regulation of nervous system development. The arcuate nucleus (ARC) of the hypothalamus integrates insulin signals and primary sensory information about circulating nutrients (e.g., glucose) to coordinate the neuroendocrine system and maintain glucose homeostasis (51). Thus, imbalances in the nervous system may disrupt glucose metabolism. The results of our KEGG analysis showed that alterations of tight junctions were a key pathway alteration in T2DM and COVID-19 convalescence. The tight junctions of the intestinal mucosa have an important role in maintaining the permeability and integrity of the intestinal mucosa. Dysfunction of the intestinal mucosal barrier is closely related to the development of diabetes, and some studies suggested that maintaining the ecological balance in the intestine may be a novel approach to overcome insulin resistance (52, 53).

The results from the PPI networks and screening by two machine learning algorithms indicated that *PTRF* and *EHD1* were the most important hub DEGs. Since the T2D validation set (GSE51762) was derived from human islets and the ratio of patients to healthy controls was somewhat different from T2DM training, this may be an important difference factor for the relatively low AUC values. However, the AUC value of the ROC curve was still greater than 0.7 and the difference with the AUC value of the training model was less than 0.1, so we believed that it still had certain accuracy. Polymerase I and transcription release factor (*PTRF*), also known as cavin-1, is associated with caveolae (“pits”) in the plasma membrane, and functions directly in the formation and secretion of cell-derived exosomes. Most studies of *PTRF* have focused on generalized lipodystrophy (GL), and mutations in this gene are highly associated with type 4 CGL. GL is a heterogeneous congenital disease (CGL) or acquired disease (AGL) characterized by loss of adipose tissue and increased insulin resistance, and an increased predisposition to metabolic complications, such as DM, hypertriglyceridemia, and hepatic steatosis (54). Mice with *PTRF* knockout have elevated triglycerides, decreased adipose tissue mass, glucose intolerance, and hyperinsulinism (55). Although there is no definitive evidence of a mechanistic relationship of *PTRF* and T2DM, we hypothesize that downregulation of *PTRF* after COVID-19 convalescence may lead to symptoms of AGL, thus increasing the risk of T2DM. EHD1 (EH Domain Containing 1) is a protein coding gene. Diseases associated with *EHD1* include plasmacytoid cystic tumor of the pancreas and cerebral hypoplasia, neuropathy, ichthyosis, and palmoplantar keratosis syndrome. Its related pathways include Angiopoietin-like protein 8 regulatory pathway and Response to elevated platelet cytosolic Ca2+. Insulin stimulates the translocation of glucose transporter 4 (GLUT4) from a perinuclear location to the plasma membrane (56)*. EHD1* controls the normal perinuclear localization of GLUT4-containing membranes and facilitates retrograde transport of GLUT4 vesicles from early endosomes to recycling endosomes or perinuclear compartments (57, 58). This suggests that *EHD1* deficiency disrupts the insulin-regulated GLUT4 cycle in cultured adipocytes.

Our analysis of EHD1 led to the identification of eleven TFs (*HOXA5, PPARG, STAT3, KLF5, NFKB1, RELA, MAX, USF1, USF2, SREBF1, NFATC2*) and two miRNAs (hsa-mir-34a-5p, hsa-mir-26b-5p). The hsa-mir-34a-5p had been found to be highly correlated with the occurrence of T2DM caused by mixed heavy metals (59). For T2DM, hsa-mir-26b-5p was significantly down-regulated after metformin treatment (60). These substances might be suggestive of subsequent studies of diabetes after COVID-19 convalescence. We believe that the reason why the number of hub genes found in T2DM is significantly less than that in T1DM is due to the high correlation between T1DM and genetics. Studies have shown that genetic defects are the basis of T1DM. T2DM is mostly perennial onset, which is related to acquired factors and may have relatively little influence on genes. Unfortunately, we found no TFs or miRNAs related to the *PTRF* gene, possibly due to the incompleteness of the databases.

We identified T-cell and CD8 T-cell infiltration in T1DM and COVID-19 convalescence, and cytotoxic T cell infiltration in T2DM and COVID-19 convalescence were not sufficiently correlated. T1DM is an autoimmune disease in which T cells attack and destroy insulin-producing beta cells in the pancreatic islets. Effector T cells respond to pancreatic beta cell-derived peptides presented by HLA class I and II molecules, and this leads to death of beta cells and insulin deficiency (61, 62). Previous research showed that CD8 T-cell-mediated autoimmune diseases are caused by disruption of auto-reactive CD8 T-cell self-tolerance mechanisms, and that an increase in the number of auto-reactive CD8 T cells drives the transition from autoimmune progenitor cells to autoimmune mediators (63). A higher percentage of cytotoxic T cells can also occur in T2DM (64). Even so, the analysis of the characteristics of immune cells in T2DM showed that there was little correlation between T2DM and immune cells. It must be mentioned that there are certain limitations in our study. The number of public COVID-19 convalescence data sets is limited, and we did not find open PBMC data sets in the GEO database. The data sets used to find differential genes and subsequent analysis are from whole blood, which is relatively inferior in the control of variables. This cannot exclude that other components of the blood would have an effect on the exploration of its pathway. On the other hand, due to the incompleteness of the TF and miRNA databases, we did not retrieve the PTRF hub DEG that was present in T2DM and COVID-19 convalescence. In addition, we did not examine the relationships of multiple risk factors affecting diabetes, the extent of glycemic control, the presence or absence of complications, and survival rate with different molecular targets.

In summary, we used bioinformatics methods with machine learning algorithms to identify specific shared hub DEGs, potential TFs, and altered pathways that occur in DM and after COVID-19 convalescence. We also constructed and validated a diagnostic model of DM after COVID-19 convalescence. Our results provide a new point of reference for subsequent studies and also provide a basis for a new approach that could be used for prevention and management of new onset DM after COVID-19 convalescence.

## Conclusions

Our study examined biomolecules and pathways that were related to the development of new-onset DM after COVID-19 convalescence by analysis of three PBMC datasets for T1DM, three PBMC datasets for T2DM, and one whole blood dataset for COVID-19 convalescence. We also used separate datasets for model validation. The results demonstrated multiple similarities of DM and COVID-19 convalescence in terms of DEGs, TFs, miRNAs, and pathways. The results from two machine learning algorithms showed that six core DEGs were shared by T1DM and COVID-19 convalescence, and that two core DEGs were shared by T2DM and COVID-19 convalescence. We therefore consider these genes as reliable indicators of DM after COVID-19 convalescence. Our finding of the importance of these several hub DEGs suggests new directions for subsequent research, and that these molecules have potential use as therapeutic targets for patients who develop new-onset DM after COVID-19 convalescence.

## Data availability statement

The original contributions presented in the study are included in the article/**Supplementary Material**. Further inquiries can be directed to the corresponding author.

## Author contributions

JS: Data curation, Formal Analysis, Visualization, Writing – original draft, Writing – review & editing. YW: Data curation, Validation, Visualization, Writing – original draft. XD: Supervision, Writing – review & editing. SS: Funding acquisition, Supervision, Writing – review & editing.

## Supplementary material

Supplementary Figure 1
**(A) **Volcano map of differentially expressed genes in the T1DM and the healthy population datasets. **(B)** Volcano map of differentially expressed genes in the T2DM and the healthy population datasets.

Supplementary Figure 2
**(A)** curve of T1DM fold1 cross-validation **(B)** curve of T1DM fold2 cross-validation **(C)** curve of T1DM fold3 cross-validation **(D)** curve of T1DM fold4 cross-validation **(E)** curve of T1DM fold5 cross-validation.

Supplementary Figure 3
**(A)** curve of T2DM fold1 cross-validation **(B)** curve of T2DM fold2 cross-validation **(C)** curve of T2DM fold3 cross-validation **(D)** curve of T2DM fold4 cross-validation **(E)** curve of T2DM fold5 cross-validation.

Supplementary Figure 4 Nomograms for prediction of T1DM after COVID-19 convalescence **(A)** and T2DM after COVID-19 convalescence **(B)** on COVID-19 convalescence validation dataset. **(C)** ROC curves of 6 hub DEGs in the COVID-19 convalescence validation dataset. **(D)** ROC curves of 2 hub DEGs in the COVID-19 convalescence validation dataset.

Supplementary Figure 5 Correlation between EHD1 and the infiltration of different immune cells (left), and between EHD1 and immune infiltration (right).
