# Supplementary figures and images for: Combining bioinformatics and machine learning algorithms to identify and analyze shared biomarkers and pathways in COVID-19 convalescence and diabetes mellitus
Source: Front Endocrinol (Lausanne). 2023 Dec 19;14:1306325. doi: 10.3389/fendo.2023.1306325 (PMC10758397; doi:10.3389/fendo.2023.1306325)

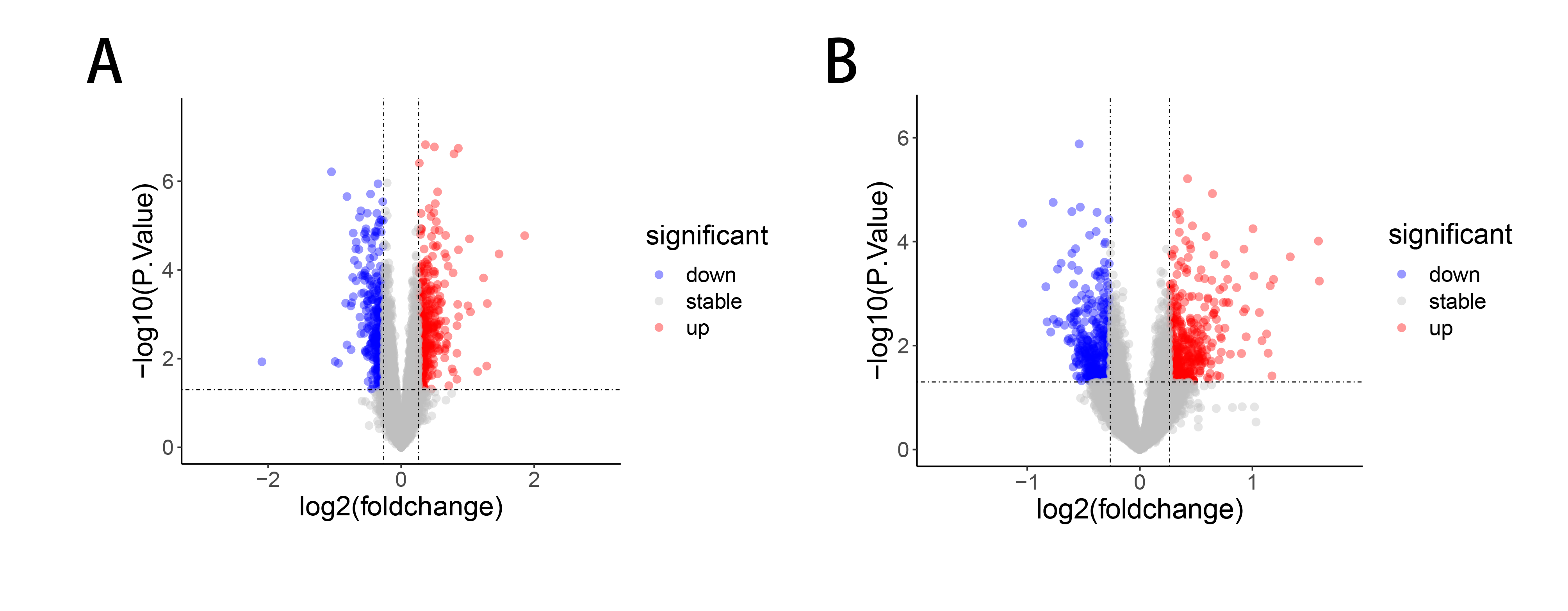

Supplement: Supplementary Figure 1 — (A) Volcano map of differentially expressed genes in the T1DM and the healthy population datasets. (B) Volcano map of differentially expressed genes in the T2DM and the healthy population datasets. [file Image_1.tif]

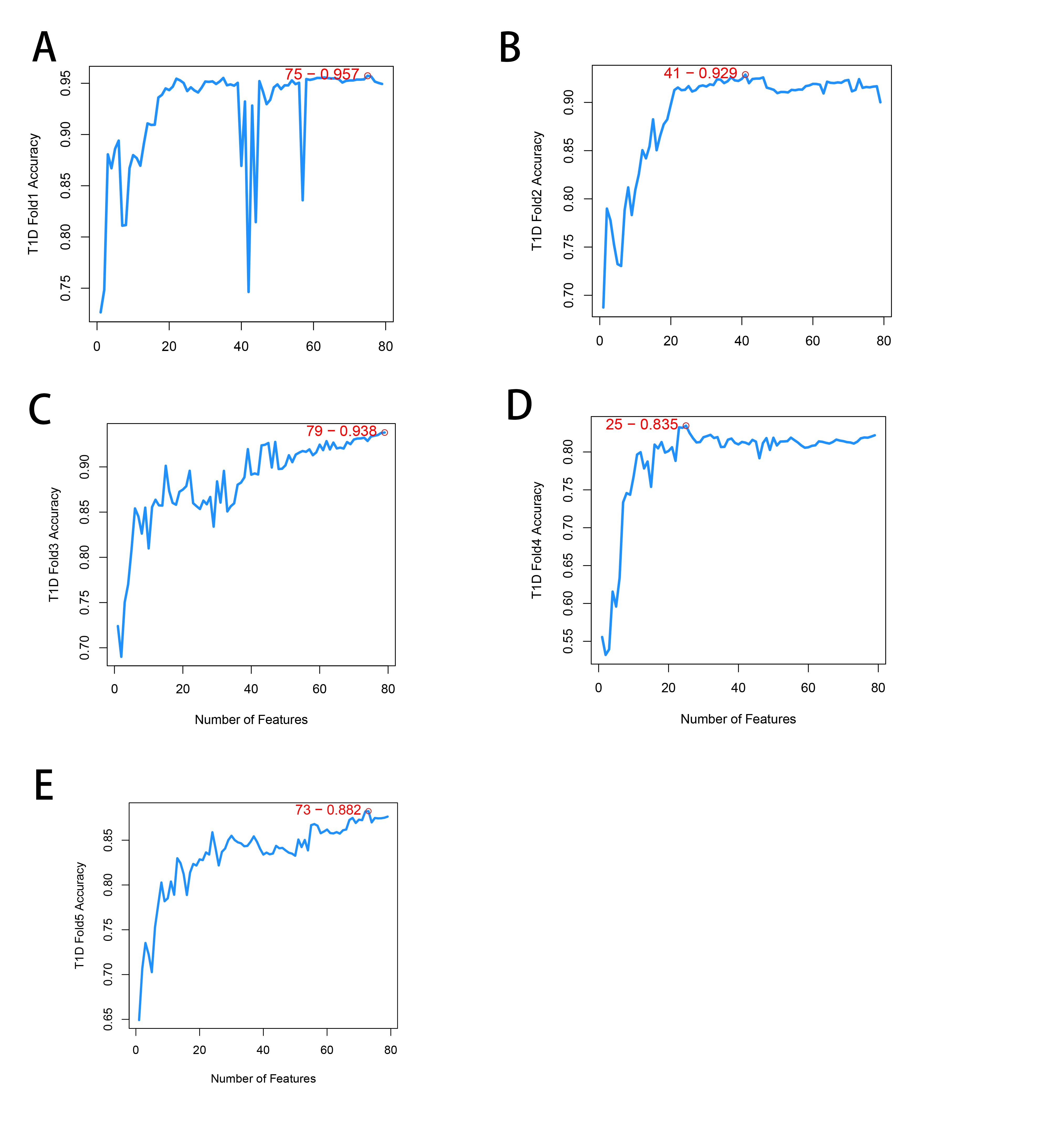

Supplement: Supplementary Figure 2 — (A) curve of T1DM fold1 cross-validation (B) curve of T1DM fold2 cross-validation (C) curve of T1DM fold3 cross-validation (D) curve of T1DM fold4 cross-validation (E) curve of T1DM fold5 cross-validation. [file Image_2.tif]

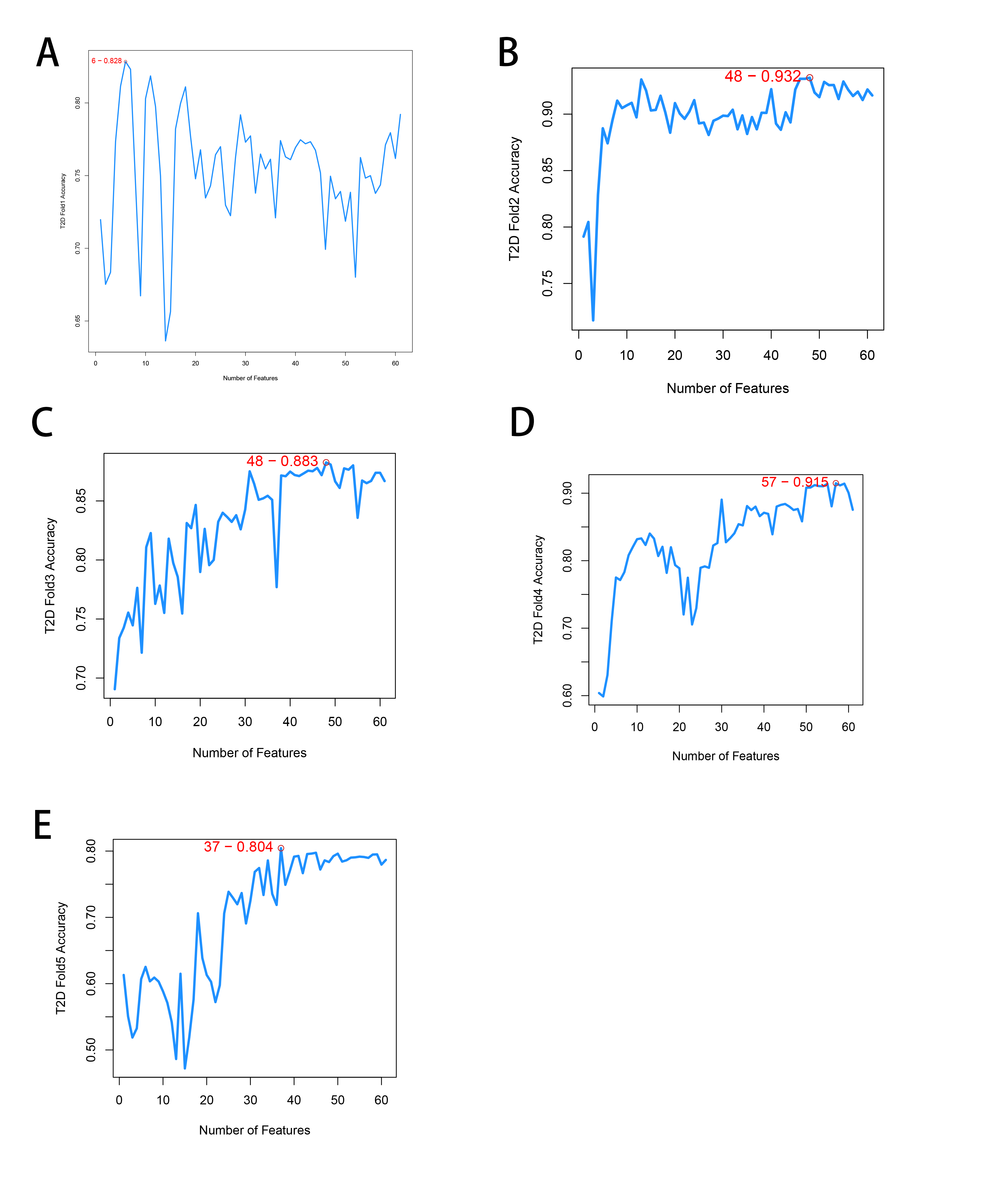

Supplement: Supplementary Figure 3 — (A) curve of T2DM fold1 cross-validation (B) curve of T2DM fold2 cross-validation (C) curve of T2DM fold3 cross-validation (D) curve of T2DM fold4 cross-validation (E) curve of T2DM fold5 cross-validation. [file Image_3.tif]

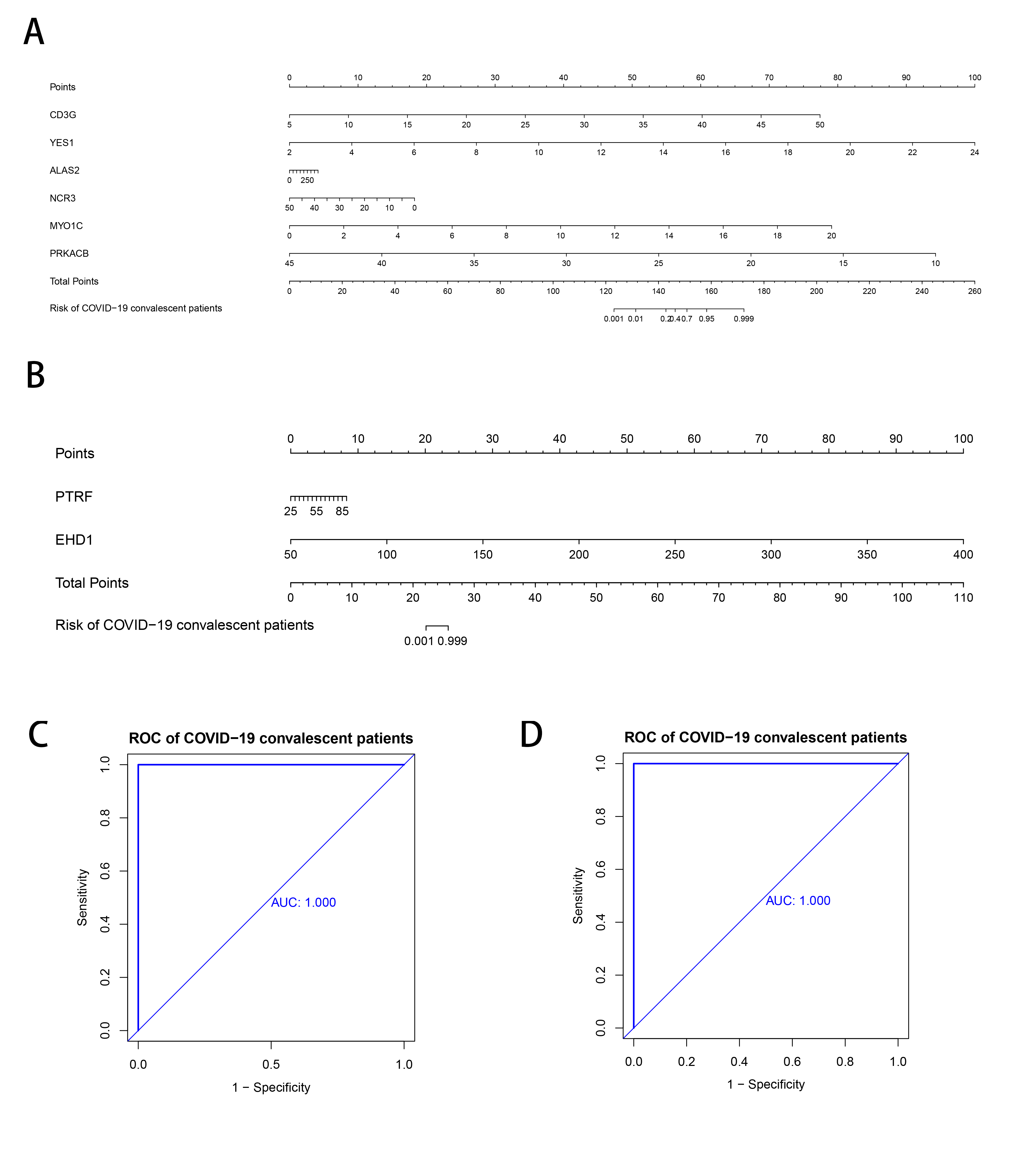

Supplement: Supplementary Figure 4 — Nomograms for prediction of T1DM after COVID-19 convalescence (A) and T2DM after COVID-19 convalescence (B) on COVID-19 convalescence validation dataset. (C) ROC curves of 6 hub DEGs in the COVID-19 convalescence validation dataset. (D) ROC curves of 2 hub DEGs in the COVID-19 convalescence validation dataset. [file Image_4.tif]

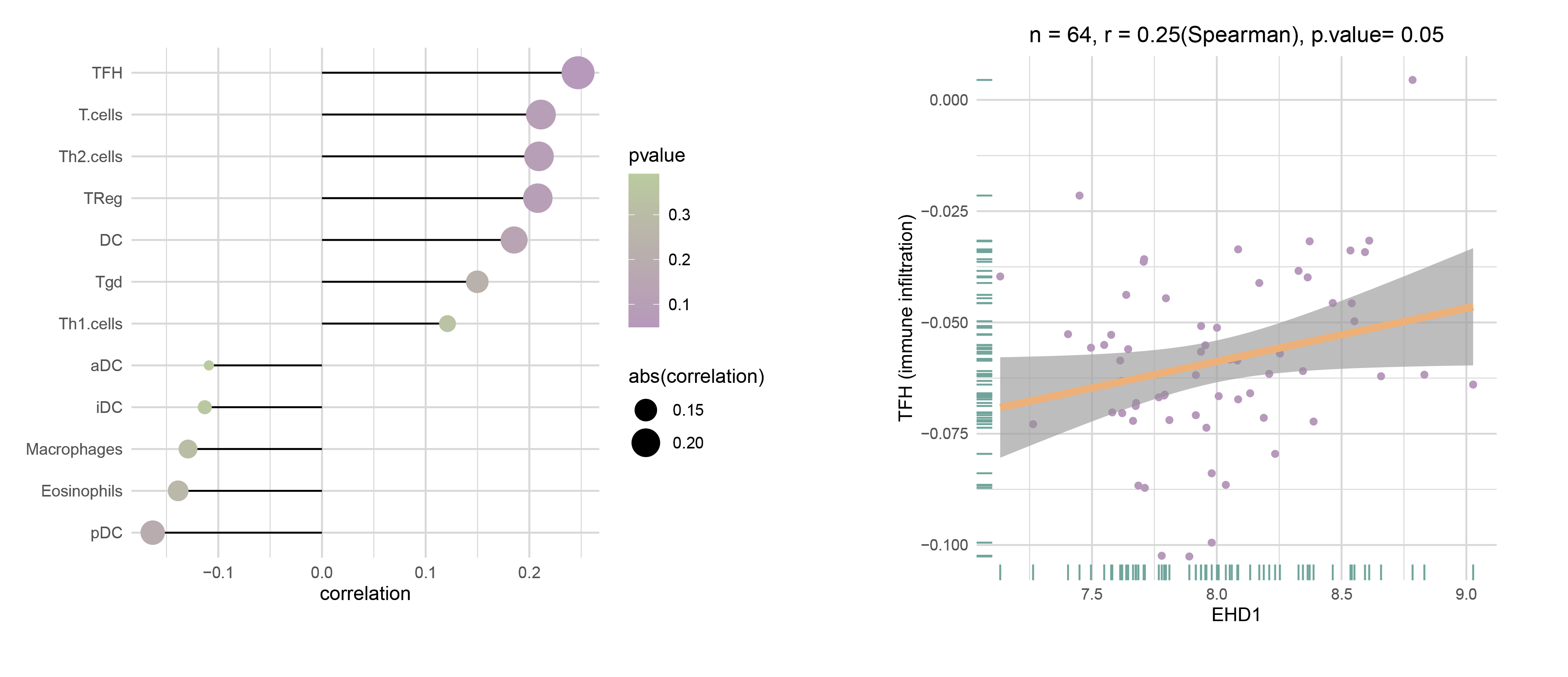

Supplement: Supplementary Figure 5 — Correlation between EHD1 and the infiltration of different immune cells (left), and between EHD1 and immune infiltration (right). [file Image_5.tif]
