# Supplementary material for: Combining bioinformatics and machine learning algorithms to identify and analyze shared biomarkers and pathways in COVID-19 convalescence and diabetes mellitus
Source: Front Endocrinol (Lausanne). 2023 Dec 19;14:1306325. doi: 10.3389/fendo.2023.1306325 (PMC10758397; doi:10.3389/fendo.2023.1306325)
Supplement: Supplementary file 6 [file Table_1.docx]

**Supplementary Table 1.** **overlapping DEGs between T1 and T2DM**

| **overlapping DEGs** | **symbol** |
| --- | --- |
| **up** | **ZNF573\|DEFA4\|LTF\|PNP\|OLFM4\|NUSAP1\|CEACAM8\|CTSG\|**  **ELANE\|TLR7\|E2F2\|CEACAM6\|MOSPD2\|CAMP\|ERCC4\|**  **EPHB3\|KCNJ2\|NFIB\|KCNJ15\|ARG1\|MS4A1\|NPL\|SLPI\|FZD3\|**  **CDC42EP3\|TCN1\|METTL13\|FZD5\|HSPA1A\|TREML2\|HBD\|**  **MPO\|NAIP\|LPAR1\|DYSF\|MX1\|ALAS2\|AQP9\|** |
| **down** | **C8orf33\|GZMM\|ZBTB16\|ID2\|SH2D2A\|CD7\|SERPINF1\|ETS1\|**  **NCR1\|IRF2BP1\|ZFP36L2\|SMPD3\|SIK1\|TNFRSF21\|TPM2\|**  **MLC1\|COL6A2\|MLLT3\|ZBTB10\|EIF5A\|JUN\|** |

**Supplementary Table 2.** Representative genes for each category.

| **Term** | **Gene symbol** |
| --- | --- |
| leukocyte activation involved in immune response  cell activation involved in immune response  immune response-activating signal transduction  immune response-regulating cell surface receptor signaling pathway  regulation of body fluid levels  activation of immune response  hemoglobin metabolic process  iron ion homeostasis  interleukin-1 beta production  positive regulation of T cell differentiation  transition metal ion homeostasis  interleukin-18 production  regulation of inflammatory response  T cell differentiation  immune response-activating cell surface receptor signaling pathway  immune response-regulating cell surface receptor signaling pathway  activation of immune response  lymphocyte differentiation  regulation of neuron projection development  extrinsic apoptotic signaling pathway via death domain receptors  extrinsic apoptotic signaling pathway  cell recognition  cellular carbohydrate metabolic process  steroid metabolic process  regulation of neuron projection development  regulation of body fluid levels  cellular cation homeostasis  Rho protein signal transduction  organic acid transport  Ras protein signal transduction  regulation of inflammatory response  cellular metal ion homeostasis  signal release  neutral lipid metabolic process  regulation of cellular ketone metabolic process  regulation of lipid metabolic process  regulation of neuron projection development  glucose homeostasis  carbohydrate homeostasis  Ras protein signal transduction  response to metal ion  regulation of GTPase activity  regulation of nervous system development  response to alcohol  response to food  regulation of nervous system process  cellular response to metal ion  cellular response to steroid hormone stimulus | DYSF/HLX/CR1  DYSF/HLX/CR1  SH2B2/DGKZ/CR1  SH2B2/DGKZ/CR1  NFE2/DGKZ/TLN1  SH2B2/DGKZ/CR1  MTHFR  ALAS2/SLC25A37  S1PR3/NLRP12  HLX/CR1  ALAS2/SLC25A37  NLRP12  SERPINF1/NR1D2/RORA/CAMK2N1/YES1  CAMK4/PIK3R1/RORA/CD3G  TNFRSF21/CD3G/YES1/NCR3  TNFRSF21/CD3G/YES1/NCR3  TNFRSF21/CD3G/YES1/NCR3  CAMK4/PIK3R1/RORA/CD3G  SERPINF1/FEZ1/PMP22/NRCAM  PMAIP1  FEM1B/PIK3R1/PMAIP1  TNFRSF21/NRCAM/NCR3  PPP1R2/RORA/PMAIP1  ABCD3/IDI1/RORA  CAMK1D/DGKG/EHD1/KLF4/LIMK1/XK  ADM/DGKG/F5/KLF4/SELP  KCNJ2/ALAS2/MAFG/SNCA/XK  ARHGAP6/BCL6/LIMK1/STARD8  ABCC3/CPT1A/SNCA/XK  ARHGAP6/BCL6/LIMK1/STARD8  BCL6/CASP5/KLF4/SNCA  KCNJ2/ALAS2/SNCA/XK  ADM/WLS/CPT1A/SNCA  CPT1A/DGKG/SNCA  ADM/CPT1A/SNCA  ADM/CPT1A/SNCA  SERPINF1/CDH1/ITGA6/RAP1A  SERPINF1/FABP5/SLC16A1  SERPINF1/FABP5/SLC16A1  NET1/RAP1A/RAP2C  SERPINF1/HNRNPD/CDH1  ITGA6/NET1/RAP1A  TNFRSF21/FABP5  HNRNPD/CDH1  SLC16A1  TNFRSF21/FABP5  SERPINF1/CDH1  SERPINF1/PGRMC2 |

**Supplementary Table 3. The mean accuracy along with the standard deviation**

|  | T1DM | | T2DM | |
| --- | --- | --- | --- | --- |
|  | mean | sd | mean | sd |
| fold1 | 0.9238626 | 0.05163109 | 0.7539976 | 0.03972165 |
| fold2 | 0.8914324 | 0.05371981 | 0.8983187 | 0.03508637 |
| fold3 | 0.885099 | 0.04849167 | 0.8303039 | 0.04846512 |
| fold4 | 0.7903953 | 0.06458528 | 0.8295214 | 0.07318799 |
| fold5 | 0.8338295 | 0.04336936 | 0.6983446 | 0.100308 |
| 5xCV | 0.8649237 | 0.04719923 | 0.8020972 | 0.04656721 |
